# A Case of Meningeal Hemorrhage

**Published:** 1879-06

**Authors:** G. A. Collamore

**Affiliations:** Toledo, Ohio


### A CASE OF MENINGEAL HEMORRHAGE.

By G. A. COLLAMORE, M.D., Toledo, Ohio.

Mr. ———, single, aged twenty-seven, book-keeper, of German descent, while playing billiards at a saloon on Monroe street, November 3, 1878, about 10 P.M , suddenly staggered, put his hand to the back of his head, and would have fallen if he had not been prevented by the bystanders. After lying a short time, he so far recovered as to be able to be removed to his home, and I was summoned.

I found him in bed, having, to all appearance, a severe rigor, as he was shivering with cold, which he complained of bitterly; and also, of a very severe pain at the back of his head and neck. I ascertained that he had eaten his dinner that day as usual; had not come up to supper, but had eaten some sardines and drank some cider. He had vomited freely soon after his first seizure, and also had free catharsis. He was in perfect possession of senses, and told me he had never lost consciousness. There had been no convulsive movements, and there was no history of any previous epilepsy. There was no paralysis then or subsequently. He continued to vomit while I stayed with him—two hours or more—but at last appeared to have become warm and sank into a doze. I gave him opiates mainly, in large doses, a part of which was vomited; applied hot flat irons and bottles of hot water to his feet and legs, and cloths wrung out of hot water to the back of his head and neck.

The next morning he was feverish and delirious, not violent, but wandering in speech and attempting to get out of bed; pulse not very rapid; vomiting still continuing in a less degree. There was no change in the pupils then or any time. Tuesday morning, the third day, he was free from delirium and appeared much better, the fever being diminished, less thirst, etc. At no time, when I used the thermometer, did it mark above 102°.

I was in doubt about the diagnosis, my first impression being that it must be some brain disease, from the suddenness of the attack and the intense pain at the occiput and nucha. In a day or two the question of malarial fever presented itself, from the fact of the initial chill, subsequent fever and delirium, and some remission in the symptoms by day, while the vomiting might be associated with either. The only brain affection which occurred to me as likely to produce these symptoms was embolism in a vertebral or the basilar artery, which might occasion the immediate symptoms, and which might be in a short time, perhaps, relieved by the collateral circulation. On inquiry, he told me that he had had several attacks of what he called rheumatism the past two years, and spoke of the successful treatment of his last attack by the use of salicylic acid and a liniment. His heart, however, showed no abnormality. I ascertained from his brother that he had not been well for a year or two, and that last year he had lost a large portion of his hair, and had taken a great deal of medicine lately.

During the week he continued to improve; the fever disappeared; the pulse came down to sixty or even less at times, though he still complained of pain in his head, sometimes at the forehead and sometimes at the back; pain in the lumbar region, and sleeplessness; he always felt worse at night. I accordingly gave him small doses of opium at night, by which he slept better. The other treatment consisted of quinine at first, and a solution of the bromides of potassium and ammonium and the iodide of potassium with an occasional laxative. After Wednesday he rarely had unusual thirst and never much appetite. On Sunday, the eighth day of his illness, he appeared so much better, having walked, with assistance, from the bed to a lounge, that I omitted the evening visit.

On Monday morning, about four o'clock, I was summoned in great haste, as he was thought to be dying. On arriving, I found that he had been thought to be sleeping, till some unusual noise attracted the attention of his brother in the adjoining room, who went to him and found him unable to speak and gasping for breath. I was told that breathing had seemed to stop entirely, and they thought he was dead; but he began to breathe again, and when I came in he was breathing more easily and was conscious. He said to me: "Doctor, can't you do something for me? I had rather be dead than suffer such pain." He lay on his back with both hands at the back of his head and neck, where I inferred his pain was. As I had been told that he was paralyzed a few minutes before, I asked him to put down one arm, which he did, then the other; then, at my request, he moved first one leg and then the other. He hawked from his throat twice a mouthful of tough, stringy mucus, which he wiped from his mouth with a towel in his right hand. The breathing now began to be spasmodic and intermittent, the interval between inspirations became longer and longer, and in a few minutes they ceased entirely; the heart continued to beat for a little time after respiration ceased. I am sure it was not five minutes from the time he spoke to me before life was extinct. I expressed the opinion that death was caused by intra-cranial hemorrhage, with some mental reservation, as the absence of the usual signs of apoplexy rendered the diagnosis somewhat obscure.

I now learned from Doctor Bond that the patient had been under his treatment the past summer for secondary syphilis, having had some cutaneous lesions. Treatment had been by iodide potassium and, I think, mercurials.

*Autopsy.*—An examination was made of the head alone twelve hours after death, Doctors Bond, Ridenour and Duncan being present. Rigidity well marked; face pale; partial alopecia about vertex. Veins and sinuses of dura mater loaded with black blood. On removal of brain, a large clot was found at the base of the skull, beneath the pia. Beginning at the junction of the basilar artery with the posterior cerebral, *i. e.*, the posterior border of the circle of Willis, it extended posteriorly over the pons and medulla oblongata, and into the cavity of the medulla spinalis farther than could be seen; laterally it nearly covered the pons and wholly the medulla; the basilar artery in its whole extent, and an inch or more of the vertebrals were completely imbedded in it. The clot also extended superiorly into the fourth ventricle, which was partially occupied by it; also, anteriorly, externally to the posterior communicating artery on the right side, to the fissure of

Sylvius, which it penetrated nearly to its external terminus This coagulum was composed of dark blood, firmly coagulated, and microscopically examined, it could not be determined whether it was all the result of the last hemorrhage, or partly of that of seven days previous; no difference could be found in any portion, though it is reasonable to conclude that the first attack was actually a hemorrhage of probably small amount, from the effects of which he was gradually recovering when prostrated by a second bleeding. The origin of the hemorrhage was not ascertained, but as the basilar and vertebral arteries were deepest imbeded in the effused blood, it is probable that one of them furnished it.

In this case we see presented a certain sequence of events. First, the condition of blood contamination, or dyscrasia, of secondary syphilis; second, an attack of cerebral confusion, dizziness, probably temporary unconsciousness, followed by vomiting and intense pain in back of head, which persists more or less severe for the following week. Finally, a sudden attack of unconsciousness, lasting a short time, recovery of consciousness, and death almost immediately ensuing. And all this without any paralysis, so far as I could ascertain, till the final overwhelming of the respiratory centers by the hemorrhage into the fourth ventricle.

There was no doubt as to the existence of syphilis in its secondary form, as indicated by the alopecia, some cutaneous eruptions and persistent headache, and from the history of the case as given by his previous physician. As to the nature of the first seizure, I was in doubt at first whether it was a hemorrhage or the effect of an embolism, both being equally excluded by the absence of paralysis. The vomiting, the intense pain at the back of the head and neck, the temporary delirium, rise of temperature, etc., however, all pointed rather to a lesion of the meninges; especially as embolism rarely, if ever, occurs without paralysis; while the apparent going on to recovery, after a few days, served still farther to obscure the diagnosis. There was, to my mind, some casual connection between the fact of syphilis and the fatal hemorrhage.

Van Buren (*Genito-Urinary Diseases with Syphilis*, p. 635,) makes this statement: "The arterial walls are subject to gummy infiltrations, either diffused between the coats of the artery for some length, thickening the same, and thus decreasing the calibre of the vessel, or developing as a distinct tumor in the vessel-wall. Aneurism may owe its origin to the weakness and softening of the arterial-wall by degeneration of gummy deposit, or the vessel may give way, allowing an appoplexy to occur. Any artery may suffer, but the carotids and arteries of the brain most commonly. An accurate diagnosis of these lesions has usually been made after death, as no symptoms during life are pathognomonic of their existence."

Doctor G. Eichler (*Deutsch. Archiv. f. Klin. Med.*, 1878, B. 22, H. 1,) considers the views advanced by Charcot and Bourchard, that the great majority of cerebral hemmorrhages are due to the bursting of miliary aneurisms of the small arteries. From the investigation of 300 to 400 cases, he concludes: (1) Primary, idiopathic cerebral hemorrhage is due to the bursting of miliary aneurisms of the smaller arteries; (2) the miliary aneurisms are *genuine* aneurisms; (3) they are due to a chronic endarteritis, which is identical with sclerosis; (4) they are, like arterial sclerosis, peculiar to old age [except when they occur in young persons as the result of syphilis.] The order of events is somewhat as follows: (1) Syphilis produces endarteritis, in some manner as yet unknown; (2) endarteritis, like all inflammations, is attended with effusion of plastic material within the arterial coats, chiefly within the intima; (3) this effusion projects into the interior of the vessel, thus diminishing its lumen; (4) this stenosis causes obstruction to the progress of the blood; hence, lateral pressure on the proximal side of narrowing is increased; hence, dilatation locally, *i. e.*, aneurism; (5) this dilatation of artery of necessity attenuates the coats, and, to that degree, weakens them; (6) this thinning and weakening of the arterial coats is liable, on any sudden or considerable increase of blood pressure, however induced, to result in rupture with its consequences.

Heubner, cited by Quincke (*Ziemssen*, Vol. VI., p. 396,)

differentiates syphilitic from ordinary endarteritis. The points of distinction are: The syphilitic form develops in a few months, the ordinary form requires years; the syphilitic is a local disease, having been solely observed in the basal-cerebral arteries, while the ordinary form extends over wide districts. The syphilitic form does not tend to fatty degeneration, like the ordinary form. It may appear at any age, while the ordinary form is a disease of advanced life.—*Toledo Med. and Surg. Journal.*

